# On the Asymptotic Behavior of the Fourier Coefficients of Mathieu Functions

**DOI:** 10.6028/jres.113.003

**Published:** 2008-02-01

**Authors:** Gerhard Wolf

**Affiliations:** Universität Duisburg-Essen Campus Essen, Fachbereich Mathematik D 45110 Essen, Germany

**Keywords:** asymptotic behavior, Fourier coefficients, Mathieu functions

## Abstract

The asymptotic behavior of the Fourier coefficients $A_{m}^{n}\left(q\right)$ and $B_{m}^{n}\left(q\right)$ of the periodic Mathieu functions ce*_n_*(*z*, *q*) and se*_n_*(*z*, *q*) is derived for fixed *n* and *q* (≠0), as *m* → ∞. Error bounds can be constructed for all approximations.

Foreword

Mathieu functions have many applications, especially in mathematics and physics:

- separation of variables in elliptical coordinates
- separation of variables in parabolic coordinates
- vibrations in a stretched elliptical ring membrane
- stability of a pendulum with periodically varying length
- amplitude distortion in moving-coil loudspeakers
- relativistic oscillators

These applications are mentioned in the chapter on Mathieu functions, written by Gerhard Wolf for the NIST *Digital Library of Mathematical Functions*. Professor Wolf is coauthor (with J. Meixner and F. W. Schäfke) of Mathieu Functions and Spheroidal Functions and their Mathematical Foundations, Lecture Notes in Mathematics 837, Springer-Verlag, 1980.

The DLMF is scheduled to begin service in 2009 from a NIST Web site. A hardcover book will be published also. These resources will provide a comprehensive guide to the higher mathematical functions for use by experienced scientific professionals.

The DLMF is modeled after the enormously successful but increasingly out-of-date NBS *Handbook of Mathematical Functions*, National Bureau of Standards Applied Mathematics Series 55, M. Abramowitz and I. A. Stegun (editors), 1964. The NBS handbook has sold more than 700,000 copies and is frequently cited in scientific journal articles. The need for a modern reference is being filled by NIST editors and staff, aided by the scientific content provided by approximately 50 external authors and validators

In addition to the main purpose of the DLMF, which is to provide a comprehensive and authoritative research tool, the project also seeks to guide further research in special functions. The paper that follows is an example. It provides the proofs of results that Wolf presents for the first time in §28.4(vii) of the DLMF.

Daniel W. Lozier

DLMF General Editor

NIST Mathematical and Computational Sciences Division

## 1. Introduction and Definitions

In this paper we aim to give asymptotic formulae for Fourier coefficients of the periodic solutions of Mathieu’s equation

$$w^{\prime \prime }+\left(a-2q\cos2z\right)w=0.$$ (1)

Equation (1) possesses the fundamental pair of solutions *w*_I_(*z*; *a*, *q*), *w*_II_(*z*; *a*, *q*) called basic solutions (see Ref. [1]) with

$$\left[\begin{array}{cc} w_{I}\left(0;a,q\right) & w_{\mathrm{II}}\left(0;a,q\right)\\ w_{I}^{\prime }\left(0;a,q\right) & w_{\mathrm{II}}^{\prime }\left(0;a,q\right) \end{array}\right]=\left[\begin{array}{cc} 1 & 0\\ 0 & 1 \end{array}\right].$$ (2)

Furthermore, we obtain eigenvalues and eigenfunctions of (1) for *n* = 0, 1, 2, ….

Table 1 gives their notations and properties. “Period *π*” means that the eigenfunction has the property *w*(*z* + *π*) = *w*(*z*), whereas “Antiperiod *π*” means that *w*(*z* + *π*) = −*w*(*z*). “Even parity” means *w*(−*z*) = *w*(*z*) and “Odd parity” means *w*(−*z*) = −*w*(*z*).

The Fourier coefficients satisfy the recurrence relations

$$\begin{array}{l} aA_{0}-qA_{2}=0,\;a=a_{2n}\left(q\right),A_{2m}=A_{2m}^{2n}\left(q\right),\\ \left(a-4\right)A_{2}-q\left(2A_{0}+A_{4}\right)=0,\\ \left(a-4m^{2}\right)A_{2m}-q\left(A_{2m-2}+A_{2m+2}\right)=0,\;m=2,3,4,\ldots , \end{array}$$ (3)

$$\begin{array}{l} \left(a-1-q\right)A_{1}-qA_{3}=0,\;a=a_{2n+1}\left(q\right),A_{2m+1}=A_{2m+1}^{2n+1}\left(q\right),\\ \left(a-{\left(2m+1\right)}^{2}\right)A_{2m+1}-q\left(A_{2m-1}+A_{2m+3}\right)=0,\;m=1,2,3,\ldots , \end{array}$$ (4)

$$\begin{array}{l} \left(a-1+q\right)B_{1}-qB_{3}=0,\;a=b_{2n+1}\left(q\right),B_{2m+1}=B_{2m+1}^{2n+1}\left(q\right),\\ \left(a-{\left(2m+1\right)}^{2}\right)B_{2m+1}-q\left(B_{2m-1}+B_{2m+3}\right)=0,\;m=1,2,3,\ldots , \end{array}$$ (5)

$$\begin{array}{l} \left(a-4\right)B_{2}-qB_{4}=0,\;a=b_{2n+2}\left(q\right),B_{2m+2}=B_{2m+2}^{2n+2}\left(q\right)\\ \left(a-4m^{2}\right)B_{2m}-q\left(B_{2m-2}+B_{2m+2}\right)=0,\;m=2,3,4,\ldots . \end{array}$$ (6)

In §2 we examine the asymptotic behavior of the coefficients $A_{2m}^{2n}\left(q\right)$, $A_{2m+1}^{2n+1}\left(q\right)$, $B_{2m+1}^{2n+1}\left(q\right)$, and $B_{2m+2}^{2n+2}\left(q\right)$ for fixed *n*, *q* (≠ 0), as *m* → ∞.

## 2. Asymptotic Forms

The following result will be proved:

**Proposition 1.** *For fixed n and q* ≠ 0*, as m* → ∞

$$\frac{A_{2m}^{2n}\left(q\right)}{A_{0}^{2n}\left(q\right)}=\frac{{\left(-1\right)}^{m}}{{\left(m!\right)}^{2}}{\left(\frac{q}{4}\right)}^{m}\frac{\pi \left(1+O\left(1/m\right)\right)}{w_{\mathrm{II}}\left(\frac{1}{2}\pi ;a_{2n}\left(q\right),q\right)},$$ (7)

$$\frac{A_{2m+1}^{2n+1}\left(q\right)}{A_{1}^{2n+1}\left(q\right)}=\frac{{\left(-1\right)}^{m+1}}{{\left({\left(\frac{1}{2}\right)}_{m+1}\right)}^{2}}{\left(\frac{q}{4}\right)}^{m+1}\frac{2\left(1+O\left(1/m\right)\right)}{w_{\mathrm{II}}^{\prime }\left(\frac{1}{2}\pi ;a_{2n+1}\left(q\right),q\right)},$$ (8)

$$\frac{B_{2m+1}^{2n+1}\left(q\right)}{B_{1}^{2n+1}\left(q\right)}=\frac{{\left(-1\right)}^{m}}{{\left({\left(\frac{1}{2}\right)}_{m+1}\right)}^{2}}{\left(\frac{q}{4}\right)}^{m+1}\frac{2\left(1+O\left(1/m\right)\right)}{w_{I}\left(\frac{1}{2}\pi ;b_{2n+1}\left(q\right),q\right)},$$ (9)

$$\frac{B_{2m}^{2n+2}\left(q\right)}{B_{2}^{2n+2}\left(q\right)}=\frac{{\left(-1\right)}^{m}}{{\left(m!\right)}^{2}}{\left(\frac{q}{4}\right)}^{m}\frac{q\pi \left(1+O\left(1/m\right)\right)}{w_{I}^{\prime }\left(\frac{1}{2}\pi ;b_{2n+2}\left(q\right),q\right)}.$$ (10)

*Proof of (7)*. We consider for *q* ≠ 0 the three term-recurrence relation

$$z_{m+1}=\left(1-\frac{a}{{\left(2m\right)}^{2}}\right)z_{m}-\frac{q^{2}}{16m^{2}{\left(m-1\right)}^{2}}z_{m-1},\;m=2,3,\ldots .$$ (11)

For the two independent solutions *u_m_*, *v_m_* of (11) with *u*_1_ = *a*, $u_{2}=a\left(1-\frac{1}{4}a\right)+\frac{1}{2}q^{2}$ and *v*_1_ = 1, $v_{2}=\left(1-\frac{1}{4}a\right)$, it follows from Sätze (Theorems) 1 and 3 of F. W. Schäfke [2] that

$$\begin{array}{l} \underset{m\to \infty }{\lim}u_{m}=-\left(2/\pi \right)w_{I}^{\prime }\left(\left(\pi /2\right);a,q\right),\\ \underset{m\to \infty }{\lim}v_{m}=\left(2/\pi \right)w_{\mathrm{II}}\left(\left(\pi /2\right);a,q\right). \end{array}$$ (12)

Transformation of (11) with

$$C_{2m}={\left(-1\right)}^{m-1}\frac{4^{m-1}}{q^{m-1}}{\left(\left(m-1\right)!\right)}^{2}z_{m},m=1,2,3,\ldots ,$$ (13)

yields

$$q\left(C_{2\left(m+1\right)}+C_{2\left(m-1\right)}\right)=\left(a-4m^{2}\right)C_{2m},m=2,3,\ldots .$$ (14)

We note the following special solutions of (14):

$$U_{2m}\;\text{with}\;z_{m}=u_{m},\;V_{2m}\;\text{with}\;z_{m}=v_{m}.$$ (15)

Then

$$\begin{array}{l} U_{2}=a,\;U_{4}=-\left(4/q\right)\left(a\left(1-\frac{1}{4}a\right)+\frac{1}{2}q^{2}\right),\\ V_{2}=1,\;V_{4}=-\left(4/q\right)\left(1-\frac{1}{4}a\right). \end{array}$$ (16)

Furthermore, we have

$$U_{2\left(m+1\right)}V_{2m}-V_{2\left(m+1\right)}U_{2m}=U_{4}V_{2}-V_{4}U_{2}=-2q.$$ (17)

If, now, *a* = *a*_2_*_n_*(*q*), then $w_{I}^{\prime }\left(\pi /2;a_{2n}\left(q\right),q\right)=0$ and *u_m_*→0 as *m*→∞.

Set *U*_0_ = *q*. Then for *m* = 2, 3, 4, …,

$$\begin{array}{l} aU_{0}-qU_{2}=0,\;\left(a-4\right)U_{2}-q\left(2U_{0}+U_{4}\right)=0,\\ \;q\left(U_{2\left(m+1\right)}+U_{2\left(m-1\right)}\right)=\left(a-4m^{2}\right)U_{2m}. \end{array}$$ (18)

Comparison with $A_{2m}^{2n}\left(q\right)$ of (3) shows that

$$q\frac{A_{2m}^{2n}\left(q\right)}{A_{0}^{2n}\left(q\right)}=U_{2m}.$$ (19)

Thus *U*_2_*_m_* is the minimal solution of (14), and by the substitution

$$U_{2m}={\left(-\frac{q}{4}\right)}^{m}\frac{1}{{\left(m!\right)}^{2}}\rho_{m}$$ (20)

we find that

$$\left(1-\frac{a_{2n}\left(q\right)}{{\left(2m\right)}^{2}}\right)\rho_{m}-\rho_{m-1}=\frac{q^{2}}{16}\frac{1}{m^{2}{\left(m+1\right)}^{2}}\rho_{m+1},$$ (21)

$$\rho_{m}-\rho_{m-1}=O\left(1/m^{2}\right),\;\text{and}\;\rho_{m}=k+O\left(1/m\right).$$ (22)

The constant *k* is determined with the aid of (17):

$$\begin{array}{l} U_{2\left(m+1\right)}V_{2m}-V_{2\left(m+1\right)}U_{2m}\\ ={\left(-\frac{q}{4}\right)}^{m+1}\frac{1}{{\left(\left(m+1\right)!\right)}^{2}}\rho_{m+1}{\left(-1\right)}^{m-1}\frac{4^{m-1}}{q^{m-1}}{\left(\left(m-1\right)!\right)}^{2}v_{m}\\ \;-{\left(-1\right)}^{m}\frac{4^{m}}{q^{m}}{\left(m!\right)}^{2}v_{m+1}{\left(-\frac{q}{4}\right)}^{m}\frac{1}{{\left(m!\right)}^{2}}\rho_{m}\\ ={\left(\frac{q}{4}\right)}^{2}\frac{1}{{\left(m\left(m-1\right)\right)}^{2}}\rho_{m+1}v_{m}-v_{m+1}\rho_{m}=-2q. \end{array}$$ (23)

and for m → ∞

$$\left(2k/\pi \right)w_{\mathrm{II}}\left(\frac{1}{2}\pi ;a_{2n}\left(q\right),q\right)=2q.$$ (24)

Together with (19) we obtain (7).

*Proof of (10)*. In the same way it follows, if *a* = *b*_2_*_n_*_+2_(*q*), then $w_{\mathrm{II}}\left(\frac{1}{2}\pi ;a,q\right)=0$, *v_m_* → 0 and

$$\begin{array}{l} \left(a-4\right)V_{2}-qV_{4}=0,\;q\left(V_{2\left(m+1\right)}+V_{2\left(m-1\right)}\right)=\left(a-4m^{2}\right)V_{2m},\\ m=2,3,4,\ldots . \end{array}$$ (25)

Comparison with $B_{2m}^{2n+2}\left(q\right)$ of (6) shows that

$$\frac{B_{2m}^{2n+2}\left(q\right)}{B_{2}^{2n+2}\left(q\right)}=V_{2m}.$$ (26)

Thus *V*_2_*_m_* is the minimal solution of (14), and for

$$V_{2m}={\left(-\frac{q}{4}\right)}^{m}\frac{1}{{\left(m!\right)}^{2}}\rho_{m}$$ (27)

we find again *ρ_m_* − *ρ_m_*_−1_ = *O*(1/*m*^2^) and

$$\rho_{m}=k+O\left(1/m\right).$$ (28)

The constant *k* can be computed via (17):

$$\begin{array}{l} U_{2\left(m+1\right)}V_{2m}-V_{2\left(m+1\right)}U_{2m}\\ =u_{m+1}\rho_{m}-{\left(\frac{q}{4}\right)}^{2}\frac{1}{{\left(m\left(m+1\right)\right)}^{2}}\rho_{m+1}u_{m}\\ =-2q. \end{array}$$ (29)

Letting *m* → ∞, we obtain

$$k=\frac{q\pi }{w_{I}^{\prime }\left(\frac{1}{2}\pi ;b_{2n+2}\left(q\right),q\right)}.$$ (30)

Together with (26) we obtain the formula (10).

*Proof of (8) and (9)*. We start with the recurrence relations

$$\begin{array}{l} z_{m+1}=\left(1-\frac{a}{{\left(2m+1\right)}^{2}}\right)z_{m}\\ \;-\frac{q^{2}}{16{\left(m+\frac{1}{2}\right)}^{2}{\left(m-\frac{1}{2}\right)}^{2}}z_{m-1},\;m=1,2,3,\ldots . \end{array}$$ (31)

The two independent solutions *u_m_* and *v_m_* of (31) with *u*_0_ = 1, *u*_1_ = *q* − *a* + 1 and *v*_0_ = 1, *v*_1_ = −*q* − *a* + 1, respectively, satisfy

$$\begin{array}{l} \underset{m\to \infty }{\lim}u_{m}=w_{I}\left(\pi /2;a,q\right),\\ \underset{m\to \infty }{\lim}v_{m}=w_{\mathrm{II}}^{\prime }\left(\pi /2;a,q\right); \end{array}$$ (32)

see Sätze (Theorems) 1 and 3 of F. W. Schäfke [2]). Then transformation of (31) with^1^

$$C_{2m+1}={\left(-4/q\right)}^{m}q{\left({\left(\frac{1}{2}\right)}_{m}\right)}^{2}z_{m},\;m=0,1,2,3,\ldots ,$$ (33)

yields

$$q\left(C_{2m+3}+C_{2m-1}\right)=\left(a-{\left(1+2m\right)}^{2}\right)C_{2m+1},\;m=1,2,3,\ldots .$$ (34)

We note the special solutions

$$U_{2m+1}\text{with}\;z_{m}=u_{m},\;V_{2m+1}\text{with}\;z_{m}=v_{m},$$ (35)

and obtain

$$U_{1}=q,\;U_{3}=a-1-q,\;V_{1}=q,\;V_{3}=a-1+q.$$ (36)

Furthermore, we observe that

$$U_{2m+1}V_{2m-1}-V_{2m+1}U_{2m-1}=U_{3}V_{1}-V_{3}U_{1}=-2q^{2}.$$ (37)

If, now, *a* = *a*_2_*_n_*_+1_(*q*), then *w*_I_(π/2; *a*_2_*_n_*_+1_(*q*), *q*) = 0 and *u_m_* → 0 for *m*→∞.

Comparison with $A_{2m+1}^{2n+1}\left(q\right)$ of (4) shows that

$$q\frac{A_{2m+1}^{2n+1}\left(q\right)}{A_{1}^{2n+1}\left(q\right)}=U_{2m+1}.$$ (38)

Thus *U*_2_*_m_*_+1_ is the minimal solution of (34), and with

$$U_{2m+1}={\left(-\frac{q}{4}\right)}^{m+1}\frac{1}{{\left({\left(\frac{1}{2}\right)}_{m+1}\right)}^{2}}\rho_{m}$$ (39)

we find that

$$\left(1-\frac{a_{2n+1}\left(q\right)}{{\left(2m+1\right)}^{2}}\right)\rho_{m}-\rho_{m-1}=\frac{q^{2}}{{\left(2m+1\right)}^{2}{\left(2m+3\right)}^{2}}\rho_{m+1},$$ (40)

$$\rho_{m}-\rho_{m-1}=O\left(1/m^{2}\right),\;\rho_{m}=k+O\left(1/m\right).$$ (41)

The constant *k* is determined with the aid of (37):

$$U_{2m+1}V_{2m-1}-V_{2m+1}U_{2m-1}=O\left(1/m^{2}\right)-qv_{m}\rho_{m-1}=-2q^{2}.$$ (42)

Letting *m* → ∞, we obtain

$$k=\frac{2q}{w_{\mathrm{II}}^{\prime }\left(\pi /2;a_{2n+1}\left(q\right),q\right)}.$$ (43)

Together with (38) we find (8).

The formula (9) is obtained by the transformation *q* → −*q*, *a*_2_*_n_*_+1_(−*q*) = *b*_2_*_n_*_+1_(*q*), and $A_{2m+1}^{2n+1}\left(q\right)={\left(-1\right)}^{n-m}B_{2m+1}^{2n+1}\left(q\right)$.

## 3. Improvement of the Rate of Convergence

If *a*_2_*_n_* ≠ (2*m*)^2^, then for *m* = 0, 1, 2, … we transform (21) via

$$\rho_{m}=\zeta_{m}/\prod_{\kappa =1}^{m}\left(1-\frac{a_{2n}\left(q\right)}{{\left(2\kappa \right)}^{2}}\right),$$ (44)

and obtain (as in Ref. [2]) ζ*_m_* − ζ*_m_*_−1_ = *O*(1/*m*^4^), ζ*_m_* − ζ = *O*(1/*m*^3^), where $\zeta =\frac{1}{2}\pi \sin\left(\frac{1}{2}\pi \sqrt{a_{2n}\left(q\right)}\right)k$ Finally we have

$$\begin{array}{l} \frac{A_{2m}^{2n}\left(q\right)}{A_{0}^{2n}\left(q\right)}=\frac{{\left(-1\right)}^{m}}{{\left(m!\right)}^{2}}{\left(\frac{q}{4}\right)}^{m}\times \\ \frac{2\sin\left(\frac{1}{2}\pi \sqrt{a_{2n}\left(q\right)}\right)\left(1+O\left(1/m^{3}\right)\right)}{\sqrt{a_{2n}\left(q\right)}\prod_{\kappa =1}^{m}\left(1-{\left(2\kappa \right)}^{-2}a_{2n}\left(q\right)\right)w_{\mathrm{II}}\left(\frac{1}{2}\pi ;a_{2n}\left(q\right),q\right)}. \end{array}$$ (45)

In the other cases, for example (40), similar results can be obtained.

Error bounds are possible with the aid of Refs. [2] and [3].

**Remark:** *Further improvements of the rate of convergence are possible by application of the results of Ref. [3].*

## Figures and Tables

**Table 1:** Eigenvalues and eigenfunctions

Eigenvalues	Eigenfunctions	Periodicity	Parity	Fourier series
*a* = *a*_2_*_n_* (*q*)	ce_2_*_n_*(*z*, *q*)	Period *π*	Even	$\sum_{m=0}^{\infty }A_{2m}^{2n}\left(q\right)\cos\left(2m\right)z$
*a* = *a*_2_*_n_*_+1_(*q*)	ce_2_*_n_*_+1_(*z*, *q*)	Antiperiod *π*	Even	$\sum_{m=0}^{\infty }A_{2m+1}^{2n+1}\left(q\right)\cos\left(2m+1\right)z$
*a* = *b*_2_*_n_*_+1_(*q*)	se_2_*_n_*_+1_(*z*, *q*)	Antiperiod *π*	Odd	$\sum_{m=0}^{\infty }B_{2m+1}^{2n+1}\left(q\right)\sin\left(2m+1\right)z$
*a* = *b*_2_*_n_*_+2_(*q*)	se_2_*_n_*_+2_(*z*, *q*)	Period *π*	Odd	$\sum_{m=0}^{\infty }B_{2m+2}^{2n+2}\left(q\right)\sin\left(2m+2\right)z$

## Footnotes

1
${\left(\frac{1}{2}\right)}_{m}$ means the Pochhammer symbol: (*a*)*_n_* = *a*(*a* + 1)(*a* + 2) … (*a* + *n* − 1).
